# Supplementary material for: Splice-Junction-Based Mapping of Alternative Isoforms in the Human Proteome
Source: Cell Rep. Author manuscript; Available in PMC 2020 Jan 15. (PMC6961840; doi:10.1016/j.celrep.2019.11.026)
Supplement: 3 [file NIHMS1546469-supplement-3.zip › DF2/PXD009737/TestisTrypsin-109-Q8IWB6-SYSTSSPIEEDFEGIQGAFAQPQVSGEEK.pdf]

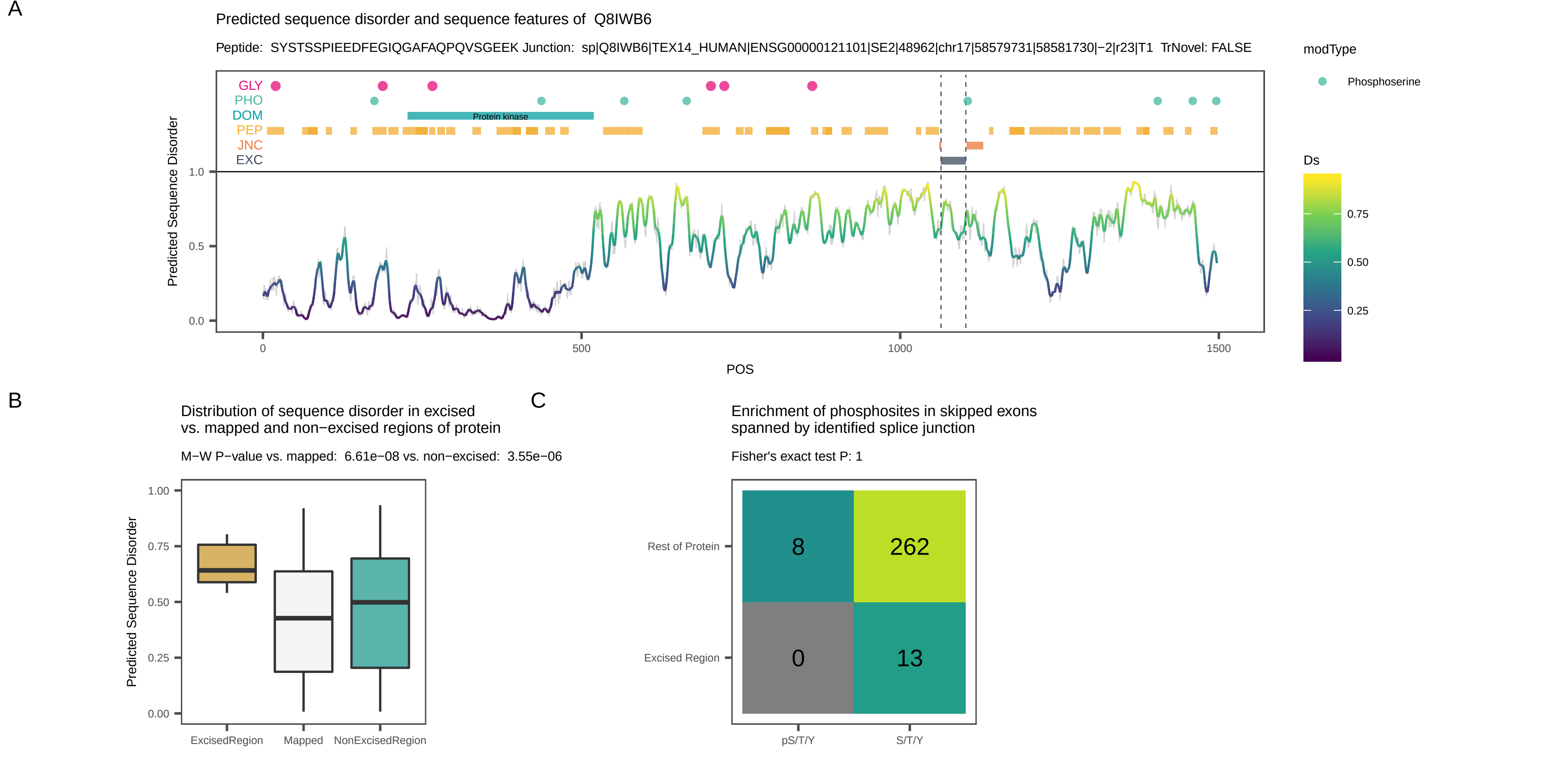

A
Predicted sequence disorder and sequence features of Q8IWB6
Peptide: SYSTSSPIEEDFEGIQGAFAQPQVSGEEK Junction: sp|Q8IWB6|TEX14_HUMAN|ENSG00000121101|SE2|48962|chr17|58579731|58581730|−2|r23|T1 TrNovel: FALSE
GLY
PHO
DOM
PEP
JNC
EXC
Protein kinase
Predicted Sequence Disorder
POS
modType
Phosphoserine
Ds
B
Distribution of sequence disorder in excised vs. mapped and non−excised regions of protein
M−W P−value vs. mapped: 6.61e−08 vs. non−excised: 3.55e−06
Predicted Sequence Disorder
ExcisedRegion
Mapped
NonExcisedRegion
C
Enrichment of phosphosites in skipped exons spanned by identified splice junction
Fisher's exact test P: 1
Rest of Protein
Excised Region
8
262
0
13
pS/T/Y
S/T/Y
